# Predicting Antileishmanial Activity of Plant-Derived Compounds Using Random Forest Modeling

**DOI:** 10.17912/micropub.biology.002261

**Published:** 2026-08-17

**Authors:** Adrianna Highgate, Patrick T. Stillson, Jandolyn Washington, Dwann Davenport

**Affiliations:** 1 Spelman College, Atlanta, GA, United States; 2 Emory University, Atlanta, GA, United States; 3 Morehouse School of Medicine, Atlanta, GA, United States; 4 Morehouse College, Atlanta, GA, United States

## Abstract

Leishmaniasis is a parasitic disease for which existing treatments can be toxic and costly, and resistance to current therapies is becoming increasingly common. This study implemented a systematic review of data regarding antileishmanial activity in plant-derived compounds and used machine learning techniques to train and test a Random Forest algorithm to predict the antileishmanial activity of plant-derived compounds. Asteraceae, Euphorbiaceae, Lamiaceae, and Myrtaceae plant families were identified for their high or moderate antileishmanial activity and nativity to areas of high leishmaniasis prevalence. The Random Forest model had 89% prediction accuracy, with an out-of-bag error rate of 16%.

**Figure 1. Identification, Classification, and Prediction of Antileishmanial Plant Compounds using Random Forest:** (A) Bioactive activity of plant families against the different clinical manifestations of leishmaniasis. In the activity analysis, the activity of a plant compound was considered "high" if the IC _50_ value was less than or equal to 10 µg/mL against the promastigote or amastigote forms. Moderate activity was defined as an IC _50_ greater than 10 µg/mL and less than 50 µg/mL, and low activity if the IC _50_ value was greater than 50 µg/mL and less than 100 µg/mL. The screens for bioactive activity were done against cutaneous leishmaniasis caused by *L. tropica* , *L. major* , *L. amazonensis* , and/or *L. panamensis* ; mucocutaneous leishmaniasis caused by *L. braziliensis* , *L. aethiopica* , *L. guyanesis* , and/or *L. panamensis* ; and visceral leishmaniasis caused by *L. infantum* and/or *L. donovani* . (B) The geographic distribution of plant species with high antileishmanial activity. (C) Random Forest model predicted antileishmanial activity with 89% accuracy.

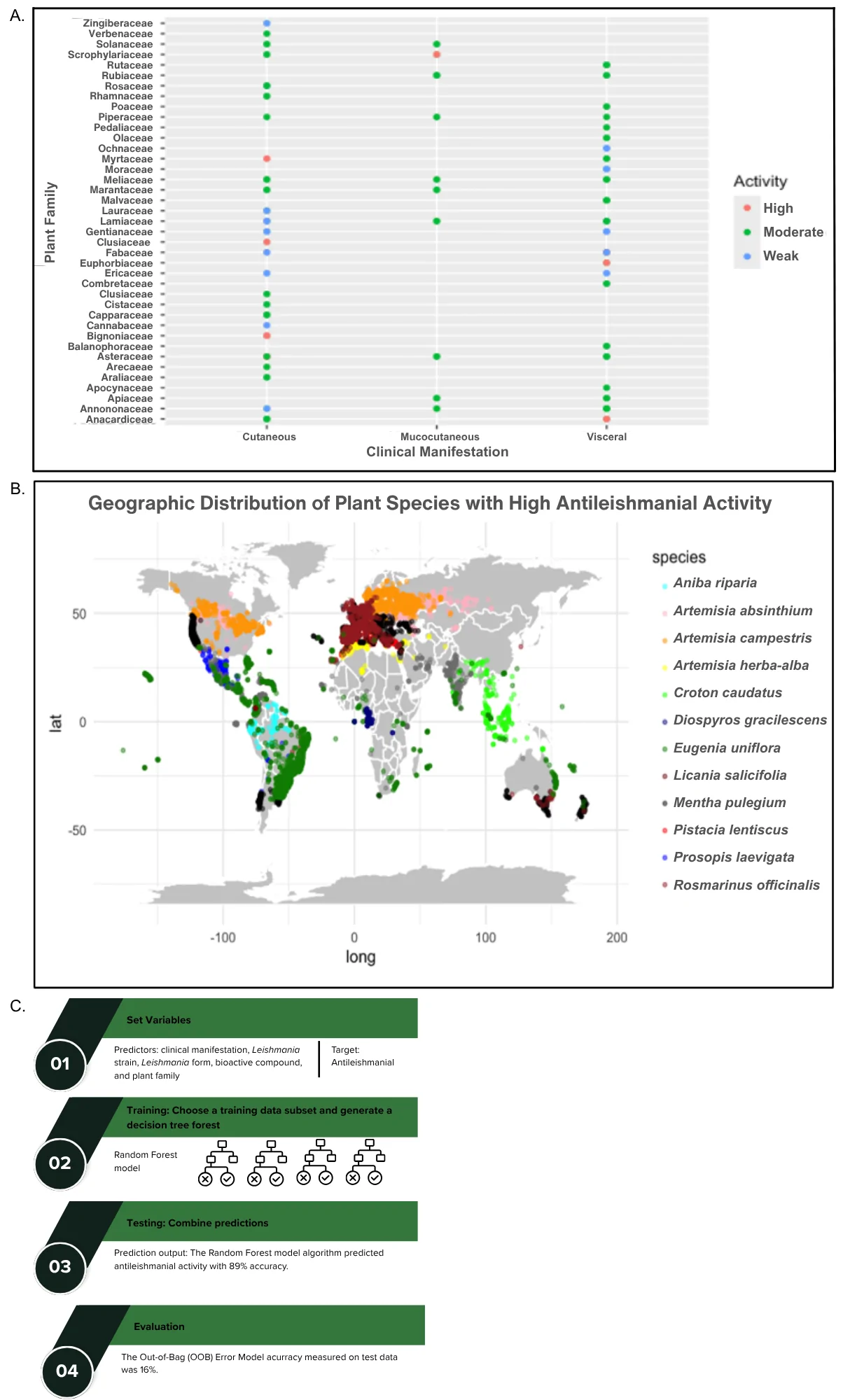



## Description

Leishmaniasis is a disease caused by protozoan parasites that spread via the bite of infected sandflies (Torres-Guerrero et al., 2017). Over 20 species of *Leishmania* have been characterized, and there are three main clinical manifestations of leishmaniasis: visceral, cutaneous, and mucocutaneous (Mann et al., 2021). Visceral leishmaniasis (VL), caused by *L. donovani* and *L. infantum* species, affects the body systemically and targets the spleen, liver, and bone marrow. VL is the most severe form of leishmaniasis and has the highest mortality and morbidity of the three forms (Mann et al., 2021). The disease burden is concentrated in East Africa, South America, and South Asia (World Health Organization, 2023). Cutaneous leishmaniasis (CL), caused by species including, but not limited to, *L. amazonensis* , *L. major* , and *L. tropica* , is characterized by skin lesions and ulcers. CL is the most common form of leishmaniasis, and cases occur primarily in the Americas, Central Asia, the Mediterranean, and the Middle East (World Health Organization, 2023). Mucocutaneous leishmaniasis (ML), caused by species including, but not limited to, *L. aethiopica* and *L. braziliensis* , deteriorates tissues in the mucous membranes of the mouth, nose, and throat (Steverding, 2017). Cases of ML occur in Bolivia, Brazil, Ethiopia, and Peru (World Health Organization, 2023).

Leishmaniasis has an estimated prevalence of 12 million cases worldwide, with 1.5-2 million new cases each year (Costa-da-Silva et al., 2022). It causes high morbidity and severe mortality (Fiuza et al., 2016). Current treatment for leishmaniasis is dependent on the clinical manifestation, *Leishmania* species, and geographic location (where treatment is administered). Liposomal amphotericin B, miltefosine, and pentavalent antimonials are the preferred treatments for VL and ML; miltefosine, paromomycin, pentavalent antimonials, and topical therapies are preferred for CL (Aronson et al., 2017; Li et al., 2026). Despite the availability of treatment options for leishmaniasis, treatment can be toxic and cost-prohibitive, and *Leishmania* resistance to many of the existing medications has become increasingly common (Li et al., 2026; Ponte-Sucre et al., 2017). Discovering additional antileishmanial compounds would contribute to efforts to overcome the limitations of current treatments (Croft et al., 2006).

Researchers look at nature as a source for new alternatives in drug development (Li et al., 2026; Oryan, 2015). Plant-derived compounds have been recognized as bioactive agents with several immunomodulatory effects (Sadeghi-Nejad et al., 2011). However, most plant-derived compounds have not been scientifically evaluated (Oryan, 2015; Yamamoto et al., 2015) and their mechanism of action is unknown (Rodrigues et al., 2015). Large scale experiments are needed to evaluate the efficacy of the large number of potential natural compounds. (Yoo et al., 2020)

Previous studies have primarily focused on in vitro experimentation to identify medicinal uses for natural compounds (Yoo et al., 2020). However, drug therapies may be identified using machine learning. One of the major applications of machine learning is to develop predictive models to assist the drug discovery process (Kore et al., 2025). This study 1) evaluated and compared plant families with antileishmanial activity against different clinical manifestations of leishmaniasis and 2) used Random Forest, a supervised machine learning approach, to predict antileishmanial activity. The approach was selected over more complex deep-learning methods because of its historic and widespread use (Atas Guvenilir & Doğan, 2023; Carracedo-Reboredo et al., 2021; Dara et al., 2022) and because it can analyze and establish relationships between multiple predictor variables (Bonifacio-Velez De Villa et al., 2025).

A systematic literature review based on the widely accepted Preferred Reporting Items for Systematic Review and Meta-analysis (PRISMA) checklist (Page et al., 2021) was conducted to identify articles to create a dataset of plant-derived compounds with antileishmanial properties. Databases searched included PubMed, PubChem, and WorldCat, yielding an initial total of over 1,500 results. Keywords used as search terms included “plant compounds” AND “leishmaniasis”; “plant compounds” AND “ *Leishmania* ”; “leishmaniasis” AND “extract”; and “ *Leishmania* ” AND “extract.” Results were screened to only include peer-reviewed and non-duplicated sources, yielding 1,200 articles. Initial exclusion criteria included a date of publication and access. Sources that were published prior to 2000 and sources that could not be accessed from research libraries were excluded, yielding 1,100 articles. Full-text review exclusions included relevance to the study and utility of content, yielding 33 articles. Data was collected from 12 of the remaining 33 articles. The plant family, genus, and species; plant bioactive compounds; Half-Maximal Inhibitory Concentration (IC _50_ ); 50% Cytotoxicity Concentration (CC _50_ ); Selectivity Index (SI); *Leishmania* species; and *Leishmania* form were used to create the dataset.

Associations between the bioactivity level of screened plant families and clinical manifestation of leishmaniasis were analyzed using the ggplot2 package (Wickham, 2016). The geographic distribution of screened plant species with high antileishmanial activity was mined using the GBIF (Global Biodiversity Information Facility) API via the rgbif package (Chamberlain et al., 2012). Occurrence records were retrieved, filtered for valid geographic coordinates, and mapped using ggplot2. All analyses were conducted in R v.4.3.2 (R Core Team, 2025).

The plant families most commonly represented in the dataset had activity against CL (26 families), followed by VL (23), and ML (10) ( Figure 1A ). Several of the plant families located in areas with high *Leishmania* prevalence, such as Asteraceae, Euphorbiaceae, Lamiaceae, and Myrtaceae, had high or moderate activity against at least one form of *Leishmania* species. The screened plant species located in areas where the disease is most prevalent were: *Aniba riparia* (Lauraceae) and *Eugenia uniflora* (Myrtaceae), densely located in Central and South America; *Croton caudatus* (Euphorbiaceae), found mainly in Southeast Asia; *Mentha pulegium* (Lamiaceae), found in South Asia; and *Artemisia herba-alba* (Asteraceae), found in Northern Africa ( Figure 1B ).

The Random Forest algorithm was applied to train a prediction model for antileishmanial activity. The dataset was analyzed by applying random trees and making associations between data points using the function randomForest (package = randomForest) (Liaw & Wiener, 2002). Highly correlated predictor variables were removed, and the predictors used in the model were clinical manifestation, *Leishmania* strain, *Leishmania* form, bioactive compound, and plant family. The number of trees was set at n = 500, and the class weight was set to (No = 1 and Yes = 0.2) with “No” weighted more to account for the close correlation between “Yes” and *Leishmania* clearance. The Random Forest model ( Figure 1C ) predicted the antileishmanial activity of the plants with 89% accuracy, with the balanced accuracy, accounting for both error rates, at 73%. The out-of-bag error, an estimate of predictive performance based on samples not used in the tree construction, was 16%.

Drug discovery is a time-consuming and costly process that involves the screening of bioactive compounds to identify potential candidates (Atas Guvenilir & Doğan, 2023). Machine learning algorithms may accelerate the drug identification process by predicting the bioactivity of plant compounds (Yabuuchi et al., 2023). This study distinguished between antileishmanial activity levels based on plant family relative to clinical manifestation; identified the geographic location of plants with high antileishmanial activity; and supported the use of machine learning to predict antileishmanial activity in plant compounds. Researchers may use machine learning to assist in determining which plants may be screened for antileishmanial activity in the lab.
